# The Importance of Pragmatic Study Design to the Scholarly Influence of Surgical Hip Fracture Randomized Controlled Trials

**DOI:** 10.5435/JAAOSGlobal-D-21-00161

**Published:** 2023-03-07

**Authors:** Meir Marmor, Guy Guenthner, Tatiana Getman, Michelle Ghert

**Affiliations:** From the Orthopaedic Trauma Institute (OTI), San Francisco General Hospital, University of California, San Francisco (UCSF), San Francisco, CA (Dr. Marmor, and Getman); Department of Orthopaedic Surgery, Tufts Medical Center, Tufts University School of Medicine, Boston, MA (Guenthner); the Western University of Health Sciences, Pomona, CA (Getman); and the McMaster University Division of Orthopaedic Surgery, Juravinski Hospital, Hamilton, ON, Canada (Dr. Ghert).

## Abstract

Surgical randomized controlled trials (RCTs) have potential drawbacks, leading some to question their role in filling the information gap in orthopaedic surgery. Pragmatism in study design was introduced to increase the clinical applicability of study results. The purpose of this study was to examine how pragmatism affects the scholarly influence of surgical RCTs.

**Methods:**

A search for surgical hip fracture-related RCTs published between 1995 and 2015 was done. Journal impact factor, citation number, research question, significance and type of outcome, number of centers involved, and the Pragmatic-Explanatory Continuum Indicator Summary-2 level of pragmatism score were recorded for each study. Scholarly influence was estimated by a study's inclusion into orthopaedic literature or guidelines or through the study's average yearly citation rate.

**Results:**

One hundred sixty RCTs were included in the final analysis. A multivariate logistic regression identified large study sample size as the only predictor of an RCT being used in clinical guidance texts. Large sample size and multicenter RCTs were predictors of high yearly citation rates. The level of pragmatism in study design did not predict scholarly influence.

**Conclusions:**

Pragmatic design is not independently associated with increased scholarly influence; however, large study sample size was the most important study characteristic affecting scholarly influence.

The primary goal of clinical research is to effectively translate clinical evidence into practice. Randomized controlled trials (RCTs) together with RCT-based systematic reviews and meta-analyses are considered the highest level of evidence for therapeutic research.^1,2^

Randomization is the best research strategy to reduce bias in clinical research by virtue of its ability to distribute known and unknown confounding variables equally between treatment groups.^2-5^ Since their introduction, RCTs have been increasing in quantity and quality,^6^ with more than 40,000 RCTs currently being reported annually.^7^

RCTs also have some notable drawbacks that limit their use. They can take several years to complete and are expensive to perform.^8-10^ Furthermore, the results of RCTs may not be generalizable because of strict inclusion criteria, which limit the amount of patients who can be enrolled in a study.^9^ Limits on patient enrollment can also result from studying a rare disease or not having researcher equipoise regarding enrollment of patients into a particular arm of a RCT.^11^ Surgical RCTs have additional methodological drawbacks, such as difficulty in standardization of surgical procedures, difference in skill levels of participating surgeons, learning curves of new procedures, and various degrees of lack of blinding.^9,12-14^ A recent study found that compared with orthopaedic nonsurgical RCTs, surgical RCTs are lower in quality because of multiple factors, including difficulty in randomization and blinding.^8,15^

Because of their importance to evidence-based medicine, and despite their limitations, the orthopaedic community has been called upon to produce well conducted and adequately powered surgical RCTs that address important therapeutic issues.^8,9,16^ However, some still question if RCTs, particularly in the surgical field, should be the primary methodology to fill the information gaps in orthopaedic surgery.^17-20^

Pragmatism in clinical trials was introduced to increase the clinical applicability of their results.^21^ All trials, including RCTs, fall on a spectrum of being explanatory to being pragmatic.^21,22^ The more explanatory the RCT, the more rigorous the inclusion/exclusion criteria are, and as a result, the study is able to measure the efficacy of a treatment in a very specific population but may fail to demonstrate efficacy if the treatment was applied to the general population. The more pragmatic the RCT, the more heterogenous the study population is, and as a result, the study is able to measure the efficacy of the intervention if it was applied to broad patient groups in real-world clinical practice.^21,23^ However, the method by which more pragmatic RCTs fill knowledge gaps and inform clinical practice is not clear. The scholarly influence of a trial, as measured by its citation level and inclusion in various clinical guidelines and textbooks, may be one method by which surgical RCTs can inform clinical practice.

The purpose of this study was to examine how pragmatism affects the scholarly influence of surgical RCTs. Hip fracture surgical RCTs were used as an example.

## Methods

This systematic review was registered (PROSPERO registration number 139858) and is reported following the Preferred Reporting Items for Systematic Reviews and Meta-analyses statement.^24^ Data set is stored at the Dryad repository, DOI: 10.7272/Q6C24TPK.

### Eligibility Criteria

All surgical hip fracture-related RCTs published between 1995 and 2015 were included in this study. A surgical hip fracture-related RCT was defined as an RCT where the therapeutic intervention under investigation was surgical. The choice of this time range was to maintain relevance to contemporary clinical practice by accounting for the last 2 decades of evidence-based medicine, but also allowing time for the RCT findings to be cited and incorporated into clinical guidelines and professional society recommendations. Observational studies, biomechanics studies, preclinical studies, case reports, reviews, systematic reviews, meta-analyses, and nonsurgical RCTs were excluded.

### Search Strategy

We conducted a computerized literature search for hip fracture RCTs published between 1995 and 2015. The search was conducted on the PubMed (https://pubmed.ncbi.nlm.nih.gov/) and Embase (https://www.embase.com/) search engines.

The search algorithm for PubMed was (“fractures” [TIAB] OR “fracture” [TIAB]) AND (“hip” [TIAB] OR “trochanteric” [TIAB] OR “intertrochanteric” [TIAB] OR “inter-trochanteric” [TIAB] OR “subtrochanteric” [TIAB] OR “sub-trochanteric” [TIAB] OR “pertrochanteric” [TIAB] OR “per-trochanteric” [TIAB] OR “femur neck” [TIAB] OR “femoral neck” [TIAB] OR “proximal femur” [TIAB]) AND (“1995” [PDAT]: “2015” [PDAT]) AND (“randomized” [TIAB] OR “randomised” [TIAB]).

The search algorithm for Embase was (fractures: ti OR fracture: ti) AND (hip: ti OR intertrochanteric: ti OR trochanteric: ti OR subtrochanteric:ti OR peritrochanteric: ti OR “femur neck”: ti OR “femoral neck”: ti OR “neck of the femur”: ti) AND (randomized: ti OR randomised: ti).

### Study Selection

Abstracts of studies identified by the search strategy were reviewed by two investigators (G.G. and T.G.) independently. Eligibility criteria were applied. Disagreements were settled between the two investigators by consensus and were discussed with a third investigator (M.T.M.) when they did not reach an agreement.

### Variable Collection

Data were collected independently by a pair of reviewers (G.G., T.G.) using a predefined electronic data extraction form. Disagreements were settled between the two investigators by consensus and were discussed with a third investigator (M.T.M.) when they did not reach an agreement.

For each included article, the following parameters were collected.

1. *Study title, year of publication, primary country where research was done*
2. *Type of hip fracture studied*
3. *Type of primary research question*
4. *Journal name and impact factor* (collected using our institutional subscription to InCites Journal Citation Reports by Clarivate Analytics (https://clarivate.com/)
5. *Number of centers involved in the study* (single/multiple)
6. *Funding source* (as reported by the authors or recorded during study registration classified to for-profit, nonprofit, or both; not reported; no funding)
7. *Study registration* (classified to registered on ClinicalTrial.gov, registered on a non-American registry, or not registered).
8. *Study groups and their sample sizes*
9. *Primary outcome* (reported? yes/no, binary? Yes/no, significant difference found? Yes/no). Primary outcomes were those explicitly reported as such. If none was explicitly reported, we took the outcome as defined in the primary study objective. If the primary outcome was still not clearly identified, the article was excluded.

### Assessment of Randomized Controlled Trial Pragmatism

The Pragmatic-Explanatory Continuum Indicator Summary (PRECIS-2) score for pragmatic/explanatory design was used to calculate the degree of pragmatism for each RCT.^25^ We modified the PRECIS-2 score to exclude the “flexibility in adherence” dimension. This dimension refers to the degree of flexibility the participants have in adherence to the chosen intervention. However, in a surgical RCT, the intervention is completed during surgery and the participants have no choice but to “adhere” to it. Each of the remaining eight dimensions were given a score of 1 to 5, resulting in a maximal score (fully pragmatic study) of 40. The Modified PRECIS-2 score was scored independently by two reviewers (G.G., T.G.). Disagreements were settled between the two investigators by consensus and were discussed with a third investigator (M.T.M.) when they did not reach an agreement.

### Assessment of Scholarly Influence of a Randomized Controlled Trial

The scholarly influence of an RCT was judged by whether the study was included in clinical guidance texts and by the study citation rate. An RCT was considered as being included in clinical guidance texts if it was incorporated into recognized professional guidelines, included in recommended readings by professional societies, or included in the references in prominent professional textbooks. The American Academy of Orthopaedic Surgeons Evidence-based Clinical Practice Guidelines for management of hip fractures in the elderly, published in September 5, 2014, was used as an indication for incorporation into professional guidelines.^26^ Recommended reading lists were taken from the Orthopaedic Trauma Association website^27^ and AO Foundation surgical reference website.^28^ Inclusion in prominent professional orthopaedic trauma textbooks was assessed in the reference lists of the 2019 edition of Rockwood and Green's Fractures in Adults^29^ and 2019 edition of Skeletal Trauma.^30^ Each study's citation rate was retrieved from Google Scholar and cross-referenced with the PubMed citation rate to ensure agreement.^31^

### Statistical Analysis

Descriptive statistics (counts, percentage, means, and SD) were used to describe the data. Continuous variables such as journal impact factor, yearly citation rate and sample size were converted into categorical variables of “high” or “low.” A study was given a “high” value if its value was above the 75^th^ percentile for that variable. Binary outcomes (low versus High) were assessed using the chi-square test for categorical variables and the student’s *t*-test for continuous variables. Significance was determined after making a Bonferroni adjustment for multiple comparisons.

Linear regression was used to assess trends in the data over time. A multivariable logistic regression analysis with stepwise backward elimination and forward selection was done to identify independent variables predicting inclusion of an RCT in clinical guidance texts or having a high yearly citation level.

Statistics were calculated using a statistical package on R version 4.0.2 (2020-06-22). Copyright © 2020 The R Foundation for Statistical Computing.

## Results

**Figure 1:**
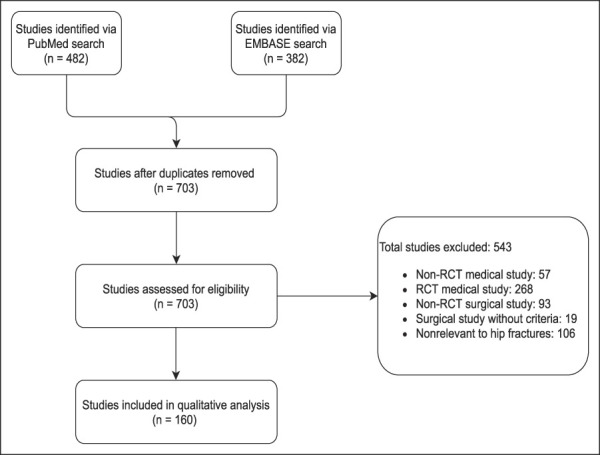
Flowchart demonstrating the manuscript review process. RCT = randomized controlled trial

**Figure 2:**
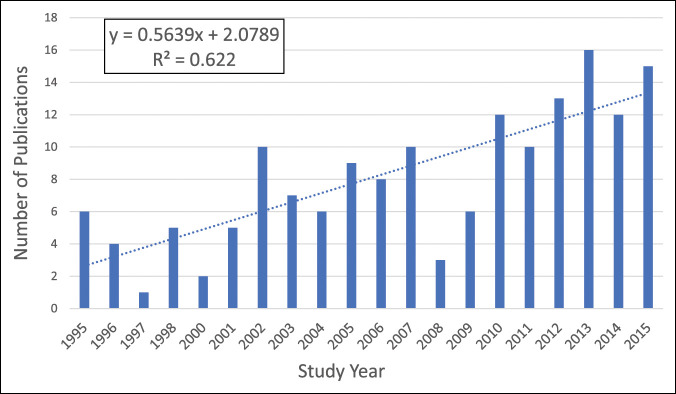
Graph showing the randomized controlled trial publication trends throughout the study period.

The initial search yielded 482 studies from PubMed and 382 studies from EMBASE. After removal of duplicates and application of eligibility criteria, 160 studies remained for final analysis (Figure 1). An upward trend (R^2^ = 0.62) was observed in the number of hip fracture RCTs published each publication year during the study period (Figure 2).

Eighty-five RCTs focused on trochanteric fractures, 73 on femoral neck fractures, two on femoral head fractures, and six on both femoral neck and trochanteric fractures. The most common research question sought to determine the best surgical implant, followed by comparing surgical techniques (Table 1). For trochanteric fractures, 37 studies (44%) focused on the question of intramedullary nail versus plate and sliding hip screw constructs. Twenty-one (13.1%) of hip fracture RCTs were multicenter trials. Thirteen studies (8%) were done in North America, nine of them (6%) in the United States. Study parameters of RCTs in relation to their inclusion in clinical guidance texts are given in Table 2. The same characteristics in relation to a high yearly citation rate are given in Table 3.

**Table 1:** Research Questions in Hip Fracture Randomized Control Trials

Factor	Both (N = 6)	Head (N = 2)	Neck (N = 72)	Trochanteric (N = 80)	Total (N = 160)
Which implant?	0 (0%)	0 (0%)	25 (34.7%)	59 (73.8%)	84 (52.5%)
Which surgical technique?	0 (0%)	0 (0%)	22 (30.6%)	11 (13.8%)	33 (20.6%)
Arthroplasty or ORIF?	0 (0%)	0 (0%)	22 (30.6%)	4 (5.0%)	26 (16.2%)
Other	6 (100%)	2 (100%)	3 (4.2%)	6 (7.5%)	17 (10.6%)

ORIF = open reduction internal fixation, RCT = randomized controlled trial, Both = femoral neck and trochanteric hip fractures, Head = femoral head fractures

**Table 2:** Study Characteristics in Relation to Their Use for Clinical Influence (Guidelines, Textbooks, Professional Websites)

Factor	Not Used (N = 95)	Used (N = 65)	Total (N = 160)	*P* ^a^
High impact factor (>4.4)				
No	78 (82.1%)	42 (64.6%)	120 (75.0%)	0.01210
Yes	17 (17.9%)	23 (35.4%)	40 (25.0%)	
Primary study location				
North America	88 (92.6%)	59 (90.8%)	147 (90.7%)	0.6720
Other	7 (7.4%)	6 (9.2%)	13 (8.0%)	
Single vs multicenter				
Multicenter	9 (9.5%)	12 (18.5%)	21 (13.1%)	0.0982
Single	86 (90.5%)	53 (81.5%)	139 (86.9%)	
Funding reported				
No	66 (69.5%)	20 (30.8%)	86 (53.8%)	1.418E-06^a^
Yes	29 (30.5%)	45 (69.2%)	74 (46.2%)	
Registration				
Clinical trials.gov	4 (4.2%)	5 (7.7%)	9 (5.6%)	0.4059
Non-American registry	3 (3.2%)	4 (6.2%)	7 (4.4%)	
Not reported	88 (92.6%)	56 (86.2%)	144 (90.0%)	
High sample size (>192)				
No	78 (82.1%)	42 (64.6%)	120 (75.0%)	0.0121
Yes	17 (17.9%)	23 (35.4%)	40 (25.0%)	
Significant primary outcome				
No	45 (47.4%)	27 (41.5%)	72 (45.0%)	0.4666
Yes	50 (52.6%)	38 (58.5%)	88 (55.0%)	
Binary primary outcome				
No	58 (61.1%)	38 (58.5%)	96 (60.0%)	0.7425
Yes	37 (38.9%)	27 (41.5%)	64 (40.0%)	
High PRECIS2 score (>42)				
No	74 (77.9%)	48 (73.8%)	122 (76.2%)	0.5545
Yes	21 (22.1%)	17 (26.2%)	38 (23.8%)	

PRECIS2 = Pragmatic-Explanatory Continuum Indicator Summary

a Statistically significant after Bonferroni adjustment for multiple comparisons.

**Table 3:** Study Characteristics in Relation to Obtaining a High Yearly Citation Average (>75th Percentile = 5 Citations Per Year on Average)

Factor	No (N = 126)	Yes (N = 34)	Total (N = 160)	*P* ^a^
High impact factor (>4.4)				
No	102 (81.0%)	18 (52.9%)	120 (75.0%)	0.0008^a^
Yes	24 (19.0%)	16 (47.1%)	40 (25.0%)	
Primary study location				
North America	115 (91.3%)	32 (94.1%)	147 (90.7%)	0.5897
Other	11 (8.7%)	2 (5.9%)	13 (8.0%)	
Single vs multicenter				
Multicenter	10 (7.9%)	11 (32.4%)	21 (13.1%)	0.0002^a^
Single	116 (92.1%)	23 (67.6%)	139 (86.9%)	
Funding reported				
No	82 (65.1%)	4 (11.8%)	86 (53.8%)	3.15E-08^a^
Yes	44 (34.9%)	30 (88.2%)	74 (46.2%)	
Registration				
Clinical trials.gov	5 (4.0%)	4 (11.8%)	9 (5.6%)	0.1829
Non-American registry	5 (4.0%)	2 (5.9%)	7 (4.4%)	
Not reported	116 (92.1%)	28 (82.4%)	144 (90.0%)	
High sample size (>192)				
No	104 (82.5%)	16 (47.1%)	120 (75.0%)	2.24E-05^a^
Yes	22 (17.5%)	18 (52.9%)	40 (25.0%)	
Significant primary outcome				
No	57 (45.2%)	15 (44.1%)	72 (45.0%)	0.9072
Yes	69 (54.8%)	19 (55.9%)	88 (55.0%)	
Binary primary outcome				
No	80 (63.5%)	16 (47.1%)	96 (60.0%)	0.0826
Yes	46 (36.5%)	18 (52.9%)	64 (40.0%)	
High PRECIS2 score (>42)				
No	96 (76.2%)	26 (76.5%)	122 (76.2%)	0.9728
Yes	30 (23.8%)	8 (23.5%)	38 (23.8%)	

PRECIS2 = Pragmatic-Explanatory Continuum Indicator Summary

a Statistically significant after Bonferroni adjustment for multiple comparisons.

Overall, we did not find a relationship between study pragmatism and increased scholarly influence. The modified PRECIS-2 scores were not found to be a predictor of both measurements of scholarly influence (inclusion in clinical guidance texts and a high average yearly citation rate). To further substantiate this finding, we conducted a sensitivity analysis by analyzing the scores as continuous variables, categorical variables, or a binary variable of whether the score was above the 75 percentile or not, did not change the results (Tables 2 and 3). In all analysis types, increased pragmatism did not correlate to increase scholarly influence.

A multivariate logistic regression analysis with stepwise backward elimination and forward selection identified only large study sample size as a statistically significant independent predictor of use of an RCT in clinical guidance texts (odds ratio [OR]: 2.5, confidence interval [CI]: 1.2 to 6.0, *P* = 0.02). Large sample size and multiple center RCTs were statistically significant predictors of high average yearly citation rates (OR: 4.7, CI: 1.9 to 11.9, *P* = 0.0008 and OR: 3.5, CI: 1.2 to 10.0, *P* = 0.017, respectively), while statistical significance in the primary outcome was suggested as a possible predictor of citation rates (OR: 2.3, CI: 0.95 to 5.8, *P* = 0.073).

## Discussion

The purpose of this study was to examine how pragmatism affects the scholarly influence of surgical RCTs. This study demonstrated that a more pragmatic design, as measured using the PRECIS-2 score, did not result in increased scholarly influence. Large sample size was the primary study characteristic that was predictive of scholarly influence. This is the first study to examine the scholarly influence of pragmatism in surgical RCT study design.

The advent of RCTs had a revolutionary effect on medical science, with more than 40,000 RCTs being reported annually.^7^ The ability of RCTs to reduce selection bias by distributing known and unknown confounding variables equally between treatment allocation groups has led them to be considered the highest level of evidence in therapeutic research.^1,2,17^ Although RCTs are more ideally suited for medical trials, surgical RCTs have been increasing in number and quality over time.^4,6^ This study demonstrated a 390% increase in the number of hip fracture RCTs between the first 3 years and the last 3 years of the study period. Higher quality hip fracture RCTs, as evidenced by the higher impact factor of the journals in which they were published, was not a significant predictor of scholarly influence in this study.

Surgical RCTs do not have a uniform clinical impact.^18^ In a previous study, a distal radius fracture RCT was shown to influence clinical practice in England by virtue of its pragmatic design.^32^ Other RCTs, may not have a similar impact on clinical practice because of conflicting results with similar RCTs or expertise bias.^18^ Measurement of clinical impact of RCTs can be difficult and can only be done adequately using a national fracture registry. The low number of hip fracture RCTs in the United States^33^ and the absence of a US national fracture registry^34^ increase this difficulty. To circumvent this difficulty, in this study, we chose to assess whether the RCTs were used for scholarly influence and not assess whether this influence was impactful. This study's findings demonstrated that it is primarily large sample size that promoted the use of an RCT for scholarly influence. This finding is in accordance with the call to publish higher quality studies with larger sample size in orthopaedic trauma.^8,16,33^ Interestingly, having a more pragmatic study design and reporting a significant primary outcome were not associated with increased scholarly influence.

The PRECIS-2 tool was developed to help quantify the level of pragmatism in a given trial.^25^ The separate domains of the PRECIS-2 tool focus on recruitment, delivery of the intervention, follow-up, and the choice of primary outcome. Therefore, trials can be deemed pragmatic about some domains, but rarely about all. As a result, challenges to designing a trial that is pragmatic in all domains have been identified.^21^ The application of the PRECIS-2 tool to the RCTs included in this study identified the fact that aspects of pragmatic design may not be consistent with important surgical outcomes. For example, many hip fracture RCTs require long-term, resource intensive follow-up to compare one surgical intervention with another. However, this degree of follow-up would be deemed “nonpragmatic” or “explanatory” because it differs from typical hip fracture follow-up. Without the ability to report important surgical outcomes, a pragmatic trial is unlikely to have scholarly or clinical impact. This highlights the challenges of the pragmatic approach in surgical trials, where trial impact may need a more explanatory approach to have impact in the field.

Citation analysis is commonly used for assessment of clinical impact and importance of research.^35,36^ A number of studies have done such an analysis on hip treatment research.^37-40^ A study on the 100 most cited publications in hip research demonstrated that only 5% of these studies were RCTs, and only 1% of studies considered “classic” were RCTs.^37^ In another study on the 50 most cited hip surgery papers, 12% of the papers were RCTs.^40^ Both of these studies suggest that, although desirable, a high level of evidence is not necessary for a study to have a high citation number. This study suggests that in order for RCTs to have a high citation rate, and ultimately a high absolute citation number, they should have a large sample size and be multicentered. Having a significant difference in the primary outcome variable trended toward statistical significance as a predictor. In two recent studies on the 50 and 100 most cited papers on hip and knee arthroplasty, respectively, most studies were published in high impact journals and originated in the United States.^38,39^ In this study, only nine of 160 RCTs (6%) originated in the United States and only 13 of 160 (8%) originated in North America.

This systematic review is the first study to examine whether a pragmatic design increases the scholarly influence of RCTs. This study also has some notable limitations. We decided to focus only on hip fracture RCTs because of their relative abundance and their applicability to many orthopaedic practices. Therefore, these findings may not be generalizable to other surgical RCTs. We did not examine the quality of the RCTs directly. Instead, we used journal impact factor as a surrogate for a high-quality paper. More detailed analysis of each study's methodology, and their specific research questions may uncover additional parameters that promote their use for scholarly influence. Finally, inclusion into clinical guidance texts, such as the American Academy of Orthopaedic Surgeons hip fracture guidelines, was determined using a limited number of texts, all of which are written in the English language and most of which originated in North America. Although these texts are widely recognized and respected, other continent-specific or country-specific texts exist and their inclusion may have influenced the results of this study.

## Conclusions

This systematic review suggests that a pragmatic design, as measured by the PRECIS-2 score, is not independently associated with increased scholarly influence. However, since large study sample size was the most important study characteristic affecting scholarly influence, a pragmatic design with broad inclusion criteria may increase scholarly influence by increasing enrollment numbers. Future studies will need to address how scholarly influence of RCTs is translated to clinical impact and whether large sample size RCTs are able to fill information gaps in orthopaedic trauma.

## References

[1] Atkins D Best D Briss PA : Grading quality of evidence and strength of recommendations. BMJ 2004;328:1490. 15205295 10.1136/bmj.328.7454.1490 PMC428525

[2] Barton S: Which clinical studies provide the best evidence? The best RCT still trumps the best observational study. BMJ 2000;321:255-256. 10915111 10.1136/bmj.321.7256.255 PMC1118259

[3] Guyatt GH Sackett DL Cook DJ: Users' guides to the medical literature. II. How to use an article about therapy or prevention. A. Are the results of the study valid? Evidence-based medicine working group. JAMA 1993;270:2598-2601. 8230645 10.1001/jama.270.21.2598

[4] Voineskos SH Coroneos CJ Ziolkowski NI : A systematic review of surgical randomized controlled trials: Part I. Risk of bias and outcomes: Common pitfalls plastic surgeons can overcome. Plast Reconstr Surg 2016;137:696-706. 26818309 10.1097/01.prs.0000475766.83901.5b

[5] Benson K Hartz AJ: A comparison of observational studies and randomized, controlled trials. N Engl J Med 2000;342:1878-1886. 10861324 10.1056/NEJM200006223422506

[6] Ahmed Ali U van der Sluis PC Issa Y : Trends in worldwide volume and methodological quality of surgical randomized controlled trials. Ann Surg 2013;258:199-207. 23774315 10.1097/SLA.0b013e31829c7795

[7] Angus DC: Fusing randomized trials with big data: The key to self-learning health care systems? JAMA 2015;314:767-768. 26305643 10.1001/jama.2015.7762

[8] Smith CS Mollon B Vannabouathong C : An assessment of randomized controlled trial quality in the Journal of Bone & Joint Surgery: Update from 2001 to 2013. J Bone Joint Surg Am 2020;102:e116. 33086352 10.2106/JBJS.18.00653

[9] McLeod RS Wright JG Solomon MJ Hu X Walters BC Lossing AL: Randomized controlled trials in surgery: Issues and problems. Surgery 1996;119:483-486. 8619200 10.1016/s0039-6060(96)80254-6

[10] Wright JG Gebhardt MC: Multicenter clinical trials in orthopaedics: Time for musculoskeletal specialty societies to take action. J Bone Joint Surg Am 2005;87:214-217. 15634834 10.2106/JBJS.D.02555

[11] Freedman B: Equipoise and the ethics of clinical research. N Engl J Med 1987;317:141-145. 3600702 10.1056/NEJM198707163170304

[12] Wallis CJD Detsky AS Fan E: Establishing the effectiveness of procedural interventions. JAMA 2018;320:2421-2422. 30383172 10.1001/jama.2018.16329

[13] Campbell AJ Bagley A Van Heest A James MA: Challenges of randomized controlled surgical trials. Orthop Clin North Am 2010;41:145-155. 20399354 10.1016/j.ocl.2009.11.001

[14] Farrokhyar F Karanicolas PJ Thoma A : Randomized controlled trials of surgical interventions. Ann Surg 2010;251:409-416. 20142732 10.1097/SLA.0b013e3181cf863d

[15] Karanicolas PJ Bhandari M Taromi B : Blinding of outcomes in trials of orthopaedic trauma: An opportunity to enhance the validity of clinical trials. J Bone Joint Surg Am 2008;90:1026-1033. 18451395 10.2106/JBJS.G.00963

[16] Bhandari M Richards RR Sprague S Schemitsch EH: The quality of reporting of randomized trials in the Journal of Bone and Joint Surgery from 1988 through 2000. J Bone Joint Surg Am 2002;84:388-396. 11886908 10.2106/00004623-200203000-00009

[17] Concato J Shah N Horwitz RI: Randomized, controlled trials, observational studies, and the hierarchy of research designs. N Engl J Med 2000;342:1887-1892. 10861325 10.1056/NEJM200006223422507 PMC1557642

[18] Harvey EJ Martineau PA Schemitsch E Nowak LL Agel J: Evidence-based medicine: Boom or bust in orthopaedic trauma? J Bone Joint Surg Am 2020;102:e6. 31609888 10.2106/JBJS.19.00547

[19] Bhandari M Giannoudis PV: Evidence-based medicine: What it is and what it is not. Injury 2006;37:302-306. 16487527 10.1016/j.injury.2006.01.034

[20] Hoppe DJ Schemitsch EH Morshed S Tornetta P Bhandari M: Hierarchy of evidence: Where observational studies fit in and why we need them. J Bone Joint Surg Am 2009;91(suppl 3):2-9. 10.2106/JBJS.H.01571 19411493

[21] Ford I Norrie J: Pragmatic trials. N Engl J Med 2016;375:454-463. 27518663 10.1056/NEJMra1510059

[22] Schwartz D Lellouch J: Explanatory and pragmatic attitudes in therapeutical trials. J Chronic Dis 1967;20:637-648. 4860352 10.1016/0021-9681(67)90041-0

[23] Bhandari M Joensson A: Clinical research for surgeons. Thieme; 2009.

[24] Moher D Liberati A Tetzlaff J Altman DG, PRISMA Group: Preferred reporting items for systematic reviews and meta-analyses: The PRISMA statement. J Clin Epidemiol 2009;62:1006-1012. 19631508 10.1016/j.jclinepi.2009.06.005

[25] Loudon K Treweek S Sullivan F Donnan P Thorpe KE Zwarenstein M: The PRECIS-2 tool: Designing trials that are fit for purpose. BMJ 2015;350:h2147. 25956159 10.1136/bmj.h2147

[26] AAOS Management of Hip Fractures in the Elderly: Evidence-Based Clinical Practice Guideline [Internet]. AAOS OrthoGuidlines, 2014. http://www.orthoguidelines.org/topic?id=1017. Accessed December 31, 2020.

[27] OTA Evidence-Based Medicine Resource List [Internet]. Orthpaedic Trauma Association. https://ota.org/education/evidence-based-medicine-resource-list. Accessed December 31, 2020.

[28] Proximal Femur [Internet]. AO Surgical Reference. https://www2.aofoundation.org/wps/portal/surgery?showPage=diagnosis&bone=Femur&segment=Proximal. Accessed December 31, 2020.

[29] Tornetta P III Ricci W McQueen MM: Rockwood and Green's fractures in adults, ed 9. Philadelphia, PA, Lippincott Williams & Wilkins, 2019.

[30] Bruce Browner Jesse Jupiter Christian Krettek Paul Anderson: Skeletal Trauma: Basic Science, Management, and Reconstruction, vol 2, ed 6. Philadelphia, PA, Elsevier, 2019, pp 3032.

[31] Google Scholar [Internet]. https://scholar.google.com/schhp?hl=en&as_sdt=0,5. Accessed January 1, 2021.

[32] Costa ML Jameson SS Reed MR: Do large pragmatic randomised trials change clinical practice? Assessing the impact of the distal radius acute fracture fixation trial (DRAFFT). Bone Joint J 2016;98-B:410-413. 26920968 10.1302/0301-620X.98B3.36730

[33] Bernstein J Weintraub S Morris T Ahn J: Randomized controlled trials for geriatric hip fracture are rare and underpowered: A systematic review and a call for greater collaboration. J Bone Joint Surg Am 2019;101:e132. 31567688 10.2106/JBJS.19.00407

[34] Carlson BC Robinson WA Wanderman NR : The American orthopaedic association's own the Bone® database: A national quality improvement project for the treatment of bone health in fragility fracture patients. Osteoporos Int 2018;29:2101-2109. 29858634 10.1007/s00198-018-4585-7

[35] Cheek J Garnham B Quan J: What's in a number? Issues in providing evidence of impact and quality of research(ers). Qual Health Res 2006;16:423-435. 16449691 10.1177/1049732305285701

[36] Adams AB Simonson D: Publication, citations, and impact factors of leading investigators in critical care medicine. Respir Care 2004;49:276-281. 15032205

[37] Ahmad SS Albers CE Büchler L : The hundred most cited publications in orthopaedic hip research–a bibliometric analysis. Hip Int 2016;26:199-208. 26951547 10.5301/hipint.5000322

[38] Holzer LA Holzer G: The 50 highest cited papers in hip and knee arthroplasty. J Arthroplasty 2014;29:1878. 24780204 10.1016/j.arth.2014.03.017

[39] Piuzzi NS Sultan AA Gatta J : Top 100 most-cited clinical studies of hip and knee arthroplasty: The foundation of practice. Orthopedics 2019;42:e151-e161. 30763450 10.3928/01477447-20190211-05

[40] Formby PM Wagner SC Mack AW: Fifty most-cited articles in the orthopaedic treatment of the hip. J Surg Orthop Adv 2016;25:165-171. 27791973

